# 1196. Determining Risk Criteria for Direct Oral Penicillin Challenge in Hospitalized Adults with a History of Penicillin Allergy

**DOI:** 10.1093/ofid/ofad500.1036

**Published:** 2023-11-27

**Authors:** Lina Hamid, Christian Bell

**Affiliations:** M Health Fairview, Minneapolis, Minnesota; M Health Fairview, Minneapolis, Minnesota

## Abstract

**Background:**

Beta (β)-lactam antibiotics such as penicillin (PCN) and its derivatives are safe and effective for many bacterial infections. PCN allergies are self-reported in approximately 10% of Americans. Concerns for allergy cross-reactivity often causes the use of broad-spectrum antibiotics and second-line agents. This may increase the cost of care, lengthen therapy, increase side effects, contribute to the development of resistance, and worsen patient outcomes. Recent studies have shown direct oral penicillin challenge (DOC) may be safe and effective in patients with low to moderate risk for reaction.

**Methods:**

This single-site retrospective analysis reviewed hospitalized adult patients with penicillin allergy assessment (PAST) completed by an antimicrobial stewardship team (AST) between May 1, 2017 and September 30, 2022 at a large academic medical center. Our primary aim is to determine risk stratification criteria for a DOC using primary literature and internal data. Secondary aims include cost avoidance analysis, updating PAST guidance, and creating an order set for the administration and monitoring of DOC.

**Results:**

A total of 256 patients were screened and 200 were included. Most common reasons for exclusion were non-PCN allergies, PAST completed at a different hospital, and PAST not completed before discharge. There were 60 patients (30%) deemed to be high-risk for allergic reaction of which 58 (96.7%) received non-PCN alternative and 2 (3.3%) received no antibiotics. AST recommended oral or IV challenge for 107 patients (53.5%) of which 34 (31.8%) received a challenge, 68 (63.6%) received non-penicillin alternative antibiotics, 3 (2.8%) were directly de-labeled, and 2 (1.9%) received no antibiotics. No anaphylactic reactions occurred in patients that received a challenge. De-labeling based on history was recommended in 33 patients (16.5%) of which 12 (36.4%) were directly de-labeled, 2 (6.1%) received a challenge, and 19 (56.1%) received non-penicillin alternative antibiotics.

**Conclusion:**

Our study confirms risk stratification for DOC is feasible and safe. Standardization of PCN allergy de-labeling with clinician and patient education may improve patient outcomes.

**Disclosures:**

**All Authors**: No reported disclosures

